# Real-world evidence of galcanezumab for migraine treatment in Japan: a retrospective analysis

**DOI:** 10.1186/s12883-022-03041-1

**Published:** 2022-12-31

**Authors:** Tsubasa Takizawa, Seiya Ohtani, Narumi Watanabe, Naoki Miyazaki, Kei Ishizuchi, Koji Sekiguchi, Chisato Iba, Mamoru Shibata, Ryo Takemura, Satoko Hori, Jin Nakahara

**Affiliations:** 1 Department of Neurology, Keio University School of Medicine, 35 Shinanomachi, Shinjuku-Ku, Tokyo, 160-8582 Japan; 2 Division of Drug Informatics, Keio University Faculty of Pharmacy, Tokyo, Japan; 3 Biostatistics Unit, Clinical and Translational Research Center, Keio University Hospital, Tokyo, Japan; 4 Department of Neurology, Tokyo Dental College Ichikawa General Hospital, Chiba, Japan

**Keywords:** Galcanezumab, Migraine, Real-world evidence, Associated symptoms, Premonitory symptoms

## Abstract

**Objective:**

To evaluate the efficacy and safety of galcanezumab in patients with migraine in a real-world setting in Japan.

**Background:**

Galcanezumab is the first anti-calcitonin gene-related peptide monoclonal antibody approved in Japan. To the best of our knowledge, no real-world studies on galcanezumab have been published in any international journal from Japan.

**Methods:**

We retrospectively examined patients with migraine who received three doses of galcanezumab between August 2021 and February 2022 at the Keio University Hospital. We assessed changes in monthly migraine days, responder rate, and migraine-associated and premonitory symptoms. We also investigated injection site reactions and adverse events.

**Results:**

Fifty-two patients received three doses of galcanezumab during the study period. Compared with those at baseline, the monthly migraine days decreased by 5.9 days (95% confidence interval, 4.2–7.7) at 3 months. The 50% responder rate was 61.5% at 3 months. A total of 64.9%, 50.0%, and 63.9% of patients showed improvement in the severity of photophobia, phonophobia, and nausea/vomiting, respectively. Premonitory symptoms without subsequent headache were reported in 62.5% of patients. Moreover, injection site reaction was the most common adverse event (34.6%).

**Conclusion:**

This study revealed the efficacy and safety of galcanezumab for migraineurs in Japan. Galcanezumab also improved migraine-associated symptoms. However, despite a reduction in headaches, premonitory symptoms without subsequent headache were reported in > 50% of the patients at 3 months.

**Supplementary Information:**

The online version contains supplementary material available at 10.1186/s12883-022-03041-1.

## Background

Migraine is a neurological disorder with a high prevalence and burden on patients [1, 2]. Migraine-preventive treatment has improved dramatically with the development of calcitonin gene-related peptide (CGRP)-targeted drugs [3].

Clinical studies have indicated the efficacy and safety of galcanezumab, an anti-CGRP monoclonal antibody, in patients with episodic migraine (EM) and chronic migraine (CM) [4, 5]. Galcanezumab is the first anti-CGRP monoclonal antibody approved in Japan (January 2021). Data on CGRPmAb clinical trials is limited for Asian populations compared to Western populations. In the pivotal global phases 2 and 3 trials of galcanezumab, Asians comprised only a small proportion of the total population [6–8]. Although clinical trials of galcanezumab focusing solely on Asians have recently been reported [4], yet there exists a need for further observing the drug’s effect among Asians population [9].

Criteria for the administration of galcanezumab differ between Japan and other countries. In Japan, galcanezumab can be used for patients with ≥ 4 migraine days per month and for those who have undergone treatment with at least one migraine-preventive drug (lomerizine, propranolol, or valproate) with ineffectiveness, intolerance, or strong concern about side effects [10]. Onabotulinumtoxin A—a globally used drug for chronic migraine—is not approved in Japan [11–14].

Real-world studies have recently reported the efficacy and safety of galcanezumab in Europe, the United States, and South Korea [9, 15–19]. However, to the best of our knowledge, no real-world studies from Japan on galcanezumab have been published in any international journal. Differences in race or criteria for the use of galcanezumab may cause differences in results between Japanese studies and those from other countries; thus, it is also necessary to study the efficacy and safety of galcanezumab in the real-world setting in Japan.

Migraineurs experience headaches and several associated symptoms (nausea, vomiting, photophobia, and phonophobia) during migraine. Moreover, migraineurs have premonitory symptoms (i.e., tiredness, stiff neck, yawning, and hunger) hours or days before the headache phase [20, 21]. To the best of our knowledge, the effects of anti-CGRP monoclonal antibodies for the treatment of non-headache symptoms have been assessed in clinical studies, but not in real-world settings [22, 23].

This study evaluated the efficacy and safety of galcanezumab in real-world settings in Japan. We also assessed the effects of galcanezumab on migraine-associated symptoms and premonitory symptoms.

## Methods

### Study design

We conducted a single-center, observational, retrospective, cohort study. The observation period was 3 months (3 M). This study was approved by the Ethics Committee of the Keio University School of Medicine (approval number: 20211144), Tokyo, Japan. Patients were informed about this observational study via the institute’s website, and they could opt out of the study. The need for informed consent was waived by the Ethics Committee of the Keio University School of Medicine, in accordance with national regulations (Ethical Guidelines for Medical and Biological Research Involving Human Subjects). All methods were carried out in accordance with relevant guidelines and regulations.

### Patients

The inclusion criteria were as follows: receipt of three doses of galcanezumab (240 mg/120 mg/120 mg) monthly from the headache group of the Keio University Hospital between August 2021 (when the drug became available at the hospital) and February 2022; fulfillment of the diagnostic criteria for migraine (including probable migraine) according to the International Classification of Headache Disorders, 3^rd^ edition (ICHD-3) [24]; and age > 18 years. Patients were diagnosed with migraine by a headache specialist (TT). Three patients were excluded from the study owing to the discontinuation of galcanezumab before completing three doses due to side effects (lightheadedness, hair loss, and eczema).

### Research items

We retrospectively collected demographic data (age, sex, height, and weight), medical history, and the following headache characteristics: age at onset, family history of headache, migraine characteristics (unilateral pain, pulsating pain, or aggravation by routine physical activity), and the presence of aura. The Generalized Anxiety Disorder-7 (GAD-7) [25, 26] and Patient Health Questionnaire-9 (PHQ-9) [27] were assessed upon administration of anti-CGRP monoclonal antibodies to determine the extent of anxiety and depression, respectively. We also collected patients’ migraine-preventive drug data, including drugs administered (lomerizine, propranolol, valproate, amitriptyline, or topiramate), use or non-use of preventive drugs at the first dosage, and handling of preventive drugs at the first dose (discontinuation or continuation).

The headache specialist (TT) explained the criteria for migraine based on the ICHD-3 to all patients, who were asked to track their headache and migraine days (including probable migraine days). Patients completed a questionnaire on the monthly migraine days (MMDs), monthly headache days (MHDs), monthly acute medication intake days (AMDs), pain intensity (0–10 Numerical Rating Scale; NRS), and associated symptoms (photophobia, phonophobia, and nausea/vomiting; none, mild, moderate, and severe) at baseline and after the first, second, and third months. The headache specialist verified the accuracy and reliability of the completed questionnaire by interviewing and occasionally reviewing each patient’s headache diary.

Patients were classified as having EM or CM according to the ICHD-3. Patients were also diagnosed with medication overuse headache (MOH) based on the ICHD-3.

Information on the injection site (forearm or abdomen), injection site reaction (pain, redness, swelling, numbness, or others) and severity (mild, moderate, or severe based on the patient’s own perspective), and other adverse reactions were also collected in the questionnaire.

Patients were asked about their satisfaction level (very satisfied, somewhat satisfied, or not satisfied) and premonitory symptoms (whether they had premonitory symptoms without subsequent headache) 3 M after receiving galcanezumab.

### Outcomes

We investigated the efficacy and safety of the therapy. As for efficacy, we investigated changes in the MMD, MHD, NRS, AMD, associated symptoms, and premonitory symptoms. We also analyzed the satisfaction level with galcanezumab administration.

The primary endpoints were a change in the MMD from baseline and 50% responder rate (RR). The 50% RR was calculated as the percentage of patients with MMD reduction from baseline by ≥ 50%. The secondary endpoints were changes from baseline in the MHD; NRS; AMD; 25%, 75%, and 100% RR; associated symptoms; premonitory symptoms; and satisfaction level with galcanezumab.

For safety, we investigated the injection site, injection site reaction, and other adverse reactions.

### Statistical analysis

Data are presented as number (percent) and mean ± standard deviation. Differences from baseline in MMD, MHD, AMD, and NRS and the least-squares means of them were analyzed using the mixed-effect model for repeated measures with time as a fixed effect and individual as a random effect, and the correlation structure was defined as unstructured. Normality was visually assessed using residual plots. The chi-squared test was used to compare the categorical data in the subgroup analysis. *P*-values for comparisons of differences from baseline were adjusted using the Bonferroni correction. We excluded missing data. All two-sided *p*-values < 0.05 were considered statistically significant. Statistical analyses were performed using SAS version 9.4 (SAS Institute Inc., Cary, NC, USA).

## Results

### Baseline characteristics

Fifty-two patients older than 18 years old were diagnosed with migraine by the headache specialist (TT) and received three doses of galcanezumab during the study period. The vast majority of the patients were women, and the mean age was 48.3 ± 12.9 (range, 19–81) years. At baseline, approximately half of the patients were classified as having EM. The mean MMD, MHD, and AMD were 12.6 ± 7.5 days/month, 15.6 ± 8.0 days/month, and 9.7 ± 7.0 days/month, respectively, and 30.8% patients were diagnosed with MOH. The NRS was 6.5 ± 1.5. Approximately half of the patients had premonitory symptoms (Table 1).

**Table 1:** Demographic and clinical characteristics of patients

**Characteristics**	EM (*n* = 25)	CM (*n* = 27)	All (*n* = 52)
**Age**, years	51.1 ± 13.3	45.7 ± 12.2	48.3 ± 12.9
**Sex**, female	23 (92.0)	23 (85.2)	46 (88.5)
**BMI,** kg/m^2^	21.3 ± 3.1	20.6 ± 2.6	21.0 ± 2.8
**Onset**, years	23.9 ± 11.9	24.2 ± 13.3	24.1 ± 12.5
**Disease history**, years	27.2 ± 17.8	21.5 ± 13.2	24.3 ± 15.7
**NRS**	6.2 ± 1.4	6.7 ± 1.5	6.5 ± 1.5
**Migraine characteristics**			
Unilateral pain	18 (72.0)	19 (70.4)	37 (71.2)
Pulsating pain	16 (64.0)	19 (70.4)	35 (67.3)
Aggravation by routine physical activity	18 (72.0)	21 (77.8)	39 (75.0)
**MMD**	7.2 ± 2.8	17.6 ± 7.2	12.6 ± 7.5
**MHD**	8.6 ± 3.8	22.0 ± 4.7	15.6 ± 8.0
**AMD**	7.0 ± 3.3	12.2 ± 8.6	9.7 ± 7.0
**Medication-overuse headache**	0 (0.0)	16 (59.3)	16 (30.8)
**Premonitory symptoms**^a^	11 (44.0)	13 (50.0)	24 (47.1)
**Aura**	5 (20.0)	7 (25.9)	12 (23.1)
**Associated symptoms**			
Photophobia	20 (80.0)	21 (77.8)	41 (78.8)
Phonophobia	16 (64.0)	22 (81.5)	38 (73.1)
Nausea/vomiting	18 (72.0)	21 (77.8)	39 (75.0)
**Medical history**			
Psychiatric	5 (20.0)	11 (40.7)	16 (30.8)
Gastrointestinal	4 (16.0)	4 (14.8)	8 (15.4)
Vascular	2 (8.0)	2 (7.4)	4 (7.7)
Hormonal	1 (4.0)	4 (14.8)	5 (9.6)
Cancer	3 (12.0)	2 (7.4)	5 (9.6)
Respiratory	4 (16.0)	4 (14.8)	8 (15.4)
Immuno—rheumatologic	2 (8.0)	3 (11.1)	5 (9.6)
Hypertension	2 (8.0)	1 (3.7)	3 (5.8)
Dyslipidemia	4 (16.0)	5 (18.5)	9 (17.3)
Diabetes mellitus	0 (0.0)	1 (3.7)	1 (1.9)
**GAD-7 ≥ 5**	11 (44.0)	15 (55.6)	26 (50.0)
**GAD-7 ≥ 10**	1 (4.0)	1 (3.7)	2 (3.8)
**PHQ-9 ≥ 5**	13 (52.0)	19 (70.4)	32 (61.5)
**PHQ-9 ≥ 10**	4 (16.0)	4 (14.8)	8 (15.4)
**Family history of headache**	9 (36.0)	16 (59.3)	25 (48.1)

Data are presented as n (%) or mean ± standard deviation

^a^We excluded one patient with CM with missing data

*EM* Episodic migraine, *CM* Chronic migraine, *BMI* Body mass index, *NRS* Numerical rating scale, *MMD* Monthly migraine day, *MHD* Monthly headache day, *AMD* Monthly acute medication intake day, *GAD-7* General anxiety disorder-7, *PHQ-9* 9-item Patient Health Questionnaire

### Preventive drugs

In terms of previous use of other migraine preventive, 30 (57.7%), 11 (21.2%), 36 (69.2%), 19 (36.5%), and 10 (19.2%) patients received lomerizine, propranolol, valproate, amitriptyline, and topiramate, respectively. Thirty-four (46.2%) patients used only one preventive drug, and the mean number of previous migraine preventives used was 2.0 ± 1.3. Twenty-five (48.1%) patients were using migraine preventives at the time of initiating galcanezumab. Three (12%) of the relevant patients discontinued consumption of migraine preventives at the first dose and 22 (88%) continued consuming them (Table 2).

**Table 2:** Preventive drugs used in studied patients

	EM (*n* = 25)	CM (*n* = 27)	All (*n* = 52)
**Types of migraine preventives**			
Lomerizine	15 (60.0)	15 (55.6)	30 (57.7)
Propranolol	6 (24.0)	5 (18.5)	11 (21.2)
Valproate	18 (72.0)	18 (66.7)	36 (69.2)
Amitriptyline	9 (36.0)	10 (37.0)	19 (36.5)
Topiramate	3 (12.0)	7 (25.9)	10 (19.2)
**Number of previous migraine preventives**			
1	9 (36.0)	15 (55.6)	24 (46.2)
2	9 (36.0)	4 (14.8)	13 (25.0)
3	4 (16.0)	3 (11.1)	7 (13.5)
≥ 4	3 (12.0)	5 (18.5)	8 (15.4)
Mean	2.0 ± 1.2	2.0 ± 1.4	2.0 ± 1.3
**Use of migraine preventives at the first dosage**			
No	9 (36.0)	18 (66.7)	27 (51.9)
Yes	16 (64.0)	9 (33.3)	25 (48.1)
Discontinued	3 (12.0)	0 (0.0)	3 (5.8)
Continued	13 (52.0)	9 (33.3)	22 (42.3)

Data are presented as n (%) or mean ± standard deviation

The migraine preventives we assessed were lomerizine, propranolol, valproate, amitriptyline, and topiramate

*EM* Episodic migraine, *CM* Chronic migraine

### Efficacy of galcanezumab for headache

#### All

**Fig.1:**
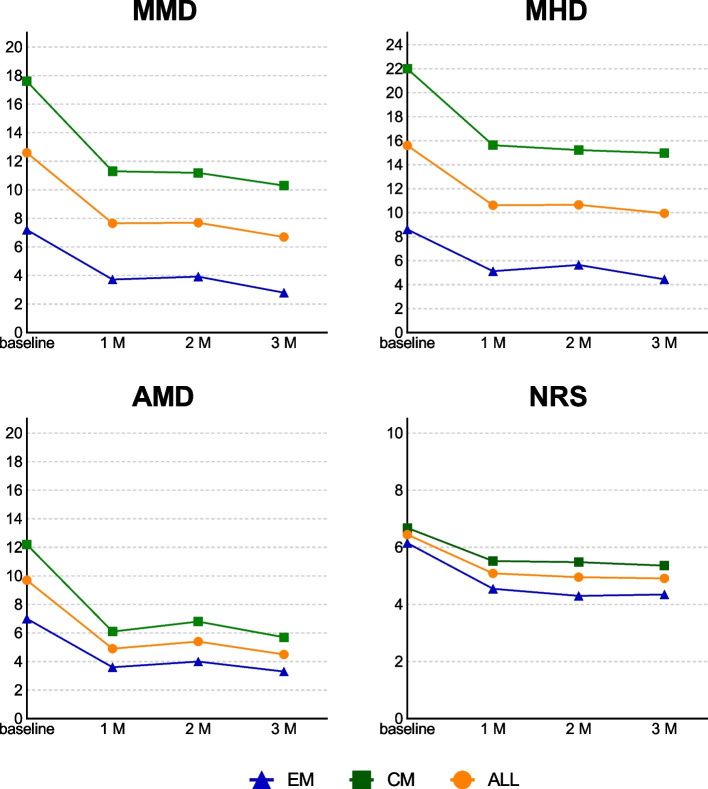
Changes in MMD, MHD, AMD, and NRS. Line graphs are expressed as mean. Blue, green, and orange lines represent EM, CM, and ALL respectively. Please note Supplementary Fig. 1 for significant change and 95% CI. MMD, monthly migraine day; MHD, monthly headache day; AMD, monthly acute medication intake days; NRS, numerical rating scale; EM, episodic migraine; CM, chronic migraine; ALL, all patients; 1 M, 1 month; 2 M, 2 months; 3 M, 3 months

At baseline, an average MMD of 12.6 ± 7.5 days/month was recorded. Compared with baseline, MMD decreased by 4.9 days (95% confidence interval [CI], 3.5–6.4; *p* < 0.001) at 1 month (1 M), 4.9 days (95% CI, 3.2–6.6; *p* < 0.001) at 2 months (2 M), and 5.9 days (95% CI, 4.2–7.7; *p* < 0.001) at 3 M). The MHD, AMD, and NRS were also significantly reduced at 1 M compared with baseline (Fig. 1, Supplementary Fig. 1).

**Fig. 2:**
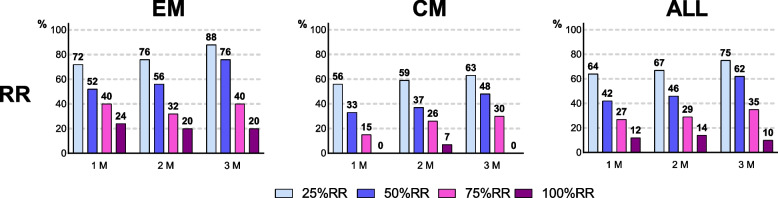
Responder rates. Proportion of patients with responder rates of 25%, 50%, 75%, and 100% based on monthly migraine days. EM, episodic migraine; CM, chronic migraine; ALL, all patients; RR, responder rate

The 50% RR was 42.3% (95% CI, 28.7–56.8) at 1 M, 46.2% (95% CI, 32.2–60.5) at 2 M, and 61.5% (95% CI, 47.0–74.7) at 3 M; a 100% RR was observed in 9.6% of patients at 3 M (Fig. 2).

### Episodic migraine

At baseline, an average MMD of 7.2 ± 2.8 days/month was recorded in those with EM. Compared with baseline, MMD decreased by 3.5 days (95% CI, 2.4–4.6; *p* < 0.001) at 1 M, 3.3 days (95% CI, 2.2–4.5; *p* < 0.001) at 2 M, and 4.4 days (95% CI, 3.4–5.4; *p* < 0.001) at 3 M. The MHD, AMD, and NRS were also significantly reduced at 1 M compared with baseline (Fig. 1, Supplementary Fig. 1).

The 50% RR was 52.0% (95% CI, 31.3–72.2) at 1 M, 56.0% (95% CI, 34.9 ~ 75.6) at 2 M, and 76.0% (95% CI, 54.9–90.6) at 3 M; a 100% RR was observed in 20.0% of patients at 3 M (Fig. 2).

### Chronic migraine

At baseline, an average MMD of 17.6 ± 7.2 days/month was recorded in those with CM. Compared with baseline, MMD decreased by 6.3 days (95% CI, 3.7–8.9; *p* < 0.001) at 1 M, 6.4 days (95% CI, 3.4–9.4; *p* < 0.001) at 2 M, and 7.3 days (95% CI, 4.0–10.6; *p* < 0.001) at 3 M. The MHD, AMD, and NRS were also significantly reduced at 1 M compared with baseline (Fig. 1, Supplementary Fig. 1).

The 50% RR was 33.3% (95% CI, 16.5–54.0) at 1 M, 37.0% (95% CI, 19.4–57.6) at 2 M, and 48.1% (95% CI, 28.7–68.1) at 3 M; a 100% RR was observed in only two patients (7.4%) at 2 M, but not at 3 M (Fig. 2).

### Patients with or without medication-overuse headache and anxiety

Supplementary Figs. 2 and 3 show the 50% RR of the subgroup analysis of MOH and non-MOH and GAD-7 ≥ 5 and GAD-7 < 5, respectively. Although the difference was not statistically significant, non-MOH patients tended to have a higher 50% RR compared to MOH patients at 3 M (*p* = 0.079). There was no tendency in the 50% RR between GAD-7 ≥ 5 and GAD-7 < 5 at 3 M.

### Associated symptoms

**Fig. 3:**
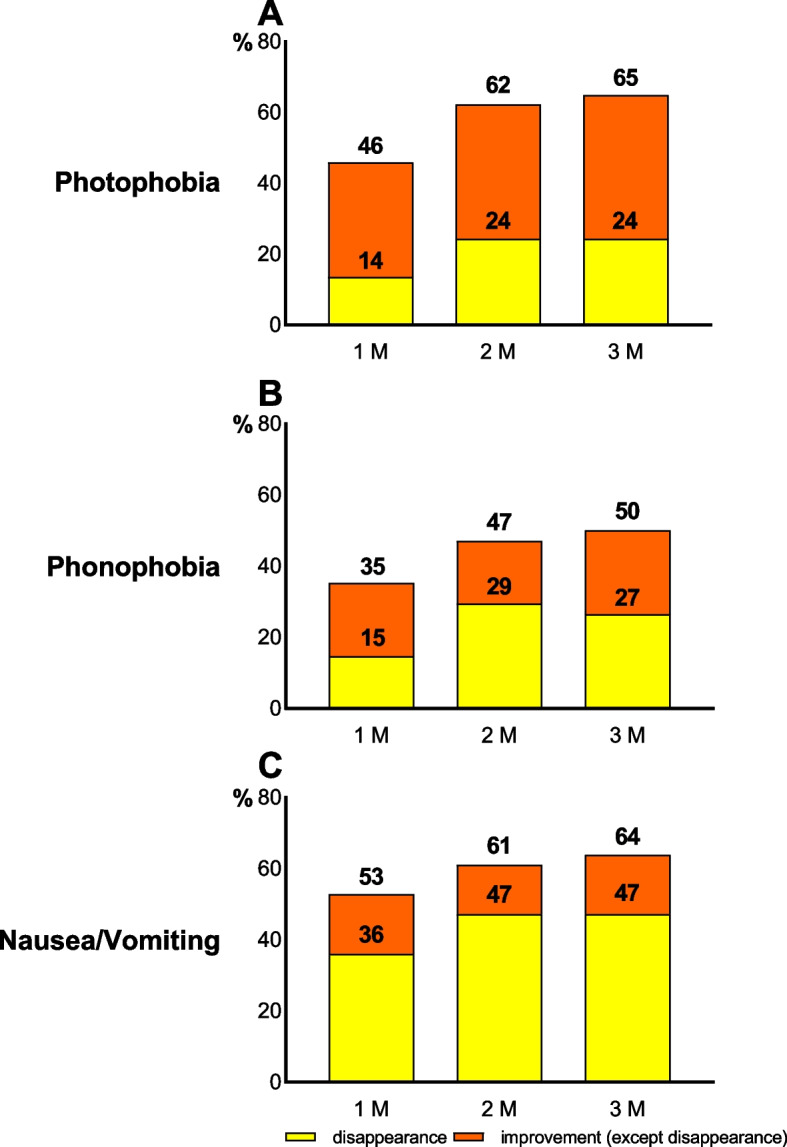
Improvement in and disappearance of the associated symptoms. (**A**) Photophobia, (**B**) Phonophobia, and (**C**) Nausea/vomiting

The severity of the associated symptoms (photophobia, phonophobia, nausea, and vomiting) at baseline and post-treatment are presented in Supplementary Fig. 4. We defined improvement in associated symptoms as a reduction in the severity of the symptoms. An improvement in photophobia was recorded in 45.9% (95% CI, 29.5–63.1), 62.2% (95% CI, 44.8–77.5), and 64.9% (95% CI, 47.5–79.8) of patients at 1 M, 2 M, and 3 M, respectively. An improvement in phonophobia was recorded in 35.3% (95% CI, 19.7–53.5), 47.1% (95% CI, 29.8–64.9), and 50.0% (95% CI, 32.4–67.6) of patients at 1 M, 2 M, and 3 M, respectively. An improvement in nausea/vomiting was recorded in 52.8% (95% CI, 35.5–69.6), 61.1% (95% CI, 43.5–76.9), and 63.9% (95% CI, 46.2–79.2) at 1 M, 2 M, and 3 M, respectively. Among patients with symptoms at baseline, photophobia, phonophobia, and nausea/vomiting disappeared in 24.3% (95% CI, 11.8–41.2), 26.5% (95% CI, 12.9–44.4), and 47.2% (95% CI, 30.4–64.5) of patients, respectively, at 3 M (Fig. 3).

Supplementary Fig. 5 shows how each associated symptom improved or disappeared in the MMD responder group (≥ 50% RR) or non-responder (< 50% RR) group at 3 M. Although the number did not reach significance in *p*-value, responders showed a higher percentage of improvement and disappearance for each associated symptom numerically compared to non-responders.

### Premonitory symptoms

Twenty-four (46.1%) patients had premonitory symptoms at baseline; among them, 15 (62.5%) reported premonitory symptoms without subsequent headache after three doses of galcanezumab.

### Safety

Most patients opted to receive the second and third dose in the upper arm (Table 3A). Fourteen (26.9%), nine (17.3%), and ten (20.0%) patients showed injection site reactions at the first, second, and third injections, respectively. During the three doses of galcanezumab, 18 (34.6%) patients had at least one episode of injection site reaction. The injection site reactions were mild to moderate, except in one patient who experienced severe redness and swelling (Table 3B). Pain was the most commonly reported injection site reaction (Table 3C).

**Table 3:** Injection site and injection site reaction after galcanezumab

(A) Injection site						
Dose	Abdomen		Forearm		n	
First	52 (100)		51 (98.1)		52	
Second	14 (27.5)		37 (72.5)		51	
Third	14 (28.0)		36 (72.0)		50	
(B) Degree of injection site reaction						
Dose	None	Mild	Moderate	Severe	n	
First	38 (73.1)	11 (21.2)	3 (5.8)	0 (0.0)	52	
Second	43 (82.7)	8 (15.4)	1 (1.9)	0 (0.0)	52	
Third	40 (80.0)	9 (18.0)	0 (0.0)	1 (2.0)	50	
(C) Types of injection site reaction						
Dose	Pain	Redness	Swelling	Numbness	Others	n
First	11 (21.2)	2 (3.8)	3 (5.8)	0 (0.0)	5 (9.6)	52
Second	7 (13.5)	4 (7.7)	4 (7.7)	0 (0.0)	1 (1.9)	52
Third	8 (16.0)	5 (10.0)	2 (4.0)	0 (0.0)	1 (2.0)	50

Data are presented as n (%)

Adverse events other than injection site reactions were constipation (*n* = 4, 7.7%), fatigue (*n* = 3, 5.8%), burning sensation (*n* = 2, 3.8%), lightheadedness (*n* = 2, 3.8%), and others (*n* = 10, 19%) (Table 4).

**Table 4:** Other adverse events

Adverse event	n (%)
Constipation	4 (7.7)
Fatigue	3 (5.8)
Burning sensation	2 (3.8)
Lightheadedness	2 (3.8)
Urticaria	1 (1.9)
Loss of hair	1 (1.9)
Stiffness in the leg	1 (1.9)
Chills	1 (1.9)
Eczema	1 (1.9)
Elevated blood pressure	1 (1.9)
Menstrual irregularity	1 (1.9)
Back pain	1 (1.9)
Somnolence	1 (1.9)
Headache	1 (1.9)

Data are presented as n (%)

### Satisfaction level

In patients who received the three doses of galcanezumab, 21 (42.0%) were very satisfied with the therapy, 20 (40.0%) were somewhat satisfied, and 9 (18.0%) were not satisfied.

## Discussion

To the best of our knowledge, this is the first, real-world study of galcanezumab and CGRPmAb in migraineurs from Japan to be reported in any international journal. Our results suggest that galcanezumab is effective and relatively safe in the Japanese population. In addition, galcanezumab improved migraine-associated symptoms, such as photophobia, phonophobia, and nausea/vomiting. The proportion of patients in whom nausea/vomiting disappeared was higher than that in whom the other two symptoms disappeared. However, premonitory symptoms remained in 62.5% of patients with 3 months of galcanezumab treatment.

The efficacy and safety of galcanezumab have been confirmed by randomized controlled trials such as EVOLVE-2 for EM and REGAIN for CM [6, 7]. In the EVOLVE-2 study on patients with EM, the change in MMD was -4.2 days/month, and the 50% RR was 59.3%. In the REGAIN study on patients with CM, the change in MMD was -4.8 days/month, and the 50% RR was 27.6%. A phase II CGAN clinical trial has evaluated the efficacy and safety of galcanezumab in Japanese patients with EM [4]. As for the efficacy, the change in MMD was -3.6 days/month, and the 50% RR was 49.8%. The efficacy (all: -5.9 MMD and 50% RR of 62.5%; EM: -4.4 MMD and 50% RR of 76.0%; and CM: -7.3 MMD and 50% RR of 48.1%) was better in this real-world study than that in the above clinical trials. The difference may be because there is no placebo arm and high expectations among patients in real-world settings.

Three real-world studies on galcanezumab from Italy, Spain, and South Korea have been published [9, 17, 18]. The study from Italy was a multicenter study, whereas the Spanish and South Korean studies were performed in single centers. CM was present in 79.8%, 87.1%, and 74.7% of patients in the studies from Italy, Spain, and South Korea, respectively. Only 51.9% of the patients in the present study had CM. In terms of failure of previous preventive drugs, the percentages of patients with ≥ 3 failures were 100% in Italy, 94.6% in Spain, and 76.7% in South Korea. Only 28.9% of patients in the present study had received ≥ 3 preventive drugs. Onabotulinumtoxin A was used in the previous studies (Italy, 45.4%; South Korea, 49.4%; Spain, 87.1%), but not in the current study. As for the 50% RR at 3 M, for high-frequency EM (HFEM), the 50% RR was 67.6%, and for CM, it was 66.7% in the Italian, 51.6% in the Spanish, and 44.8% in the South Korean study. The 50% RRs for patients in the Spanish and South Korean studies were similar to that for patients with CM in the present study (48.1%). This is probably due to the high proportion of patients with CM in the Spanish and South Korean studies. A comparison of the 50% RR for patients with HFEM in the Italian study with patients with EM in the present study suggested a higher efficacy in the present study (76.0% vs. 67.6%). This may be because the present study included 17 (68.0% with EM) patients with low-frequency EM as opposed to 0 (0%) patients in the Italian study.

All three associated symptoms improved in patients in the present study. Nausea and vomiting disappeared in a higher number of patients than photophobia and phonophobia (47.2% vs. 24.3% and 26.5%, respectively). A reason for this high rate of resolution of nausea and vomiting symptoms may be that the number of MMDs with nausea and vomiting at baseline was lower than that with photophobia and phonophobia in the previous study (EM, 4.7 ± 3.4 vs. 8.5 ± 3.5; CM, 8.8 ± 6.7 vs. 14.8 ± 7.3) [22]. However, we failed to track the types of associated symptoms during each attack; thus, we are unable to conclude whether the present cohort also showed a similar trend.

Approximately 50% of patients had premonitory symptoms at baseline. While the effects of galcanezumab were observed in some patients, 62.5% of the patients felt that the residual of premonitory symptoms even though subsequent headaches were inhibited, after three doses of galcanezumab. A post hoc analysis of data from clinical trials showed that galcanezumab reduced the frequency of migraine headache days with premonitory symptoms [19]; however, this study failed to assess days with premonitory symptoms without headache. The presence of residual premonitory symptoms is an interesting issue from the mechanistic viewpoint, that merits further studies.

In patients who received the three doses of galcanezumab, no serious adverse events were observed, and the most frequent adverse events were injection site reactions. However, three patients who were not included in the study owing to discontinuation of the therapy (side effects of lightheadedness, hair loss, or eczema) after the first or second visit contributed to a 94.5% continuation rate for 3 M. Nevertheless, the drug continuation rates for 3 M remained high. In our study, one patient had side-effect of eczema (mild), and another patient stopped galcanezumab before three doses due to eczema. Eczema has previously been reported in a patient on erenumab with asthma [28]. Interestingly, one of the two patients in our institute who developed eczema had a past history of asthma as well. Information on whether patients with allergic backgrounds develop such kind of adverse effect would be an interesting topic for future studies.

The satisfaction rate was also high at 82.0%. High satisfaction with anti-CGRP monoclonal antibodies has been reported in a real-world study [19]. This study also described satisfaction at 3 months. However, we did not include the satisfaction levels in the three patients who discontinued galcanezumab. Nevertheless, the satisfaction rate would still be high (74.5%) if we consider these patients.

This study has some strengths. It is the first real-world study from Japan that described the efficacy and safety of galcanezumab in migraineurs in detail. Moreover, we analyzed the migraine-associated and premonitory symptoms that have not been studied in a real-world setting. However, the study also has some limitations—small sample size, retrospective nature, single-center design, and a short 3-month observation period. The primary endpoint (migraine days) was mainly assessed with questionnaires and not by the actual headache diaries which were only checked in some cases. Thus, further research is necessary to elucidate the effects of anti-CGRP monoclonal antibodies in the Japanese population.

## Conclusion

This study revealed that galcanezumab is effective and safe for the prevention of migraine in Japan. Anti-CGRP monoclonal antibody use improved the migraine-associated symptoms and even resulted in disappearance of nausea and vomiting in nearly half of the patients at 3 months. However, despite a reduction in headaches, premonitory symptoms without subsequent headaches were observed in > 50% of the patients at 3 M.

## Supplementary Information

**Additional file 1: Supplementary Figure 1.** Changes in (A) MMD, (B) MHD, (C)AMD, and (D) NRS **～*** represents a statistically significant change. **adjusted *p*<0.01; ***adjusted *p*<0.001. Line graphs are expressed as mean and bars represent 95% CI. MMD, monthly migraine day; MHD, monthly headache day; AMD, monthly acute medication intake days; NRS, numerical rating scale; EM, episodic migraine; CM, chronic migraine; ALL, all patients; 1 M, 1 month; 2 M, 2 months; 3 M, 3 months.

**Additional file 2: Supplementary Figure 2.** Fifty percent responder rate in patients with or without medication-overuse headache. MOH: Medication-overuse headache. Responder rate was based on monthly migraine days.

**Additional file 3: Supplementary Figure 3.** Fifty percent responder rate in patients in GAD-7 < 5 and GAD-7 ≥ 5 (anxiety). GAD-7: Generalized Anxiety Disorder-7. Responder rate was based on monthly migraine days.

**Additional file 4: Supplementary Figure 4.** Degree of associated symptoms at baseline and after 1-3 M of galcanezumab. (A) Photophobia, (B) Phonophobia, and (C) Nausea/vomiting. 1 M: 1 month, 3 M: 3 months.

**Additional file 5: Supplementary Figure 5.** Improvement and disappearance in associated symptoms in patients with or without 50% responder rate in MMD. (A) Photophobia, (B) Phonophobia, and (C) Nausea/vomiting non-Res: non-responder (<50% responder rate), Res: responder (≥50% responder rate), MMD: monthlymigraine days. Responder rate was based on monthly migraine days.

## Data Availability

The datasets analyzed during the current study are available from the corresponding author on reasonable request.
