# Supplementary material for: Real-world evidence of galcanezumab for migraine treatment in Japan: a retrospective analysis
Source: BMC Neurol. 2022 Dec 31;22:512. doi: 10.1186/s12883-022-03041-1 (PMC9805082; doi:10.1186/s12883-022-03041-1)
Supplement: Supplementary file 1 — Additional file 1: Supplementary Figure 1. Changes in (A) MMD, (B) MHD, (C)AMD, and (D) NRS **～*** represents a statistically significant change. **adjusted p<0.01; ***adjusted p<0.001. Line graphs are expressed as mean and bars represent 95% CI. MMD, monthly migraine day; MHD, monthly headache day; AMD, monthly acute medication intake days; NRS, numerical rating scale; EM, episodic migraine; CM, chronic migraine; ALL, all patients; 1 M, 1 month; 2 M, 2 months; 3 M, 3 months. [file 12883_2022_3041_MOESM1_ESM.pdf]

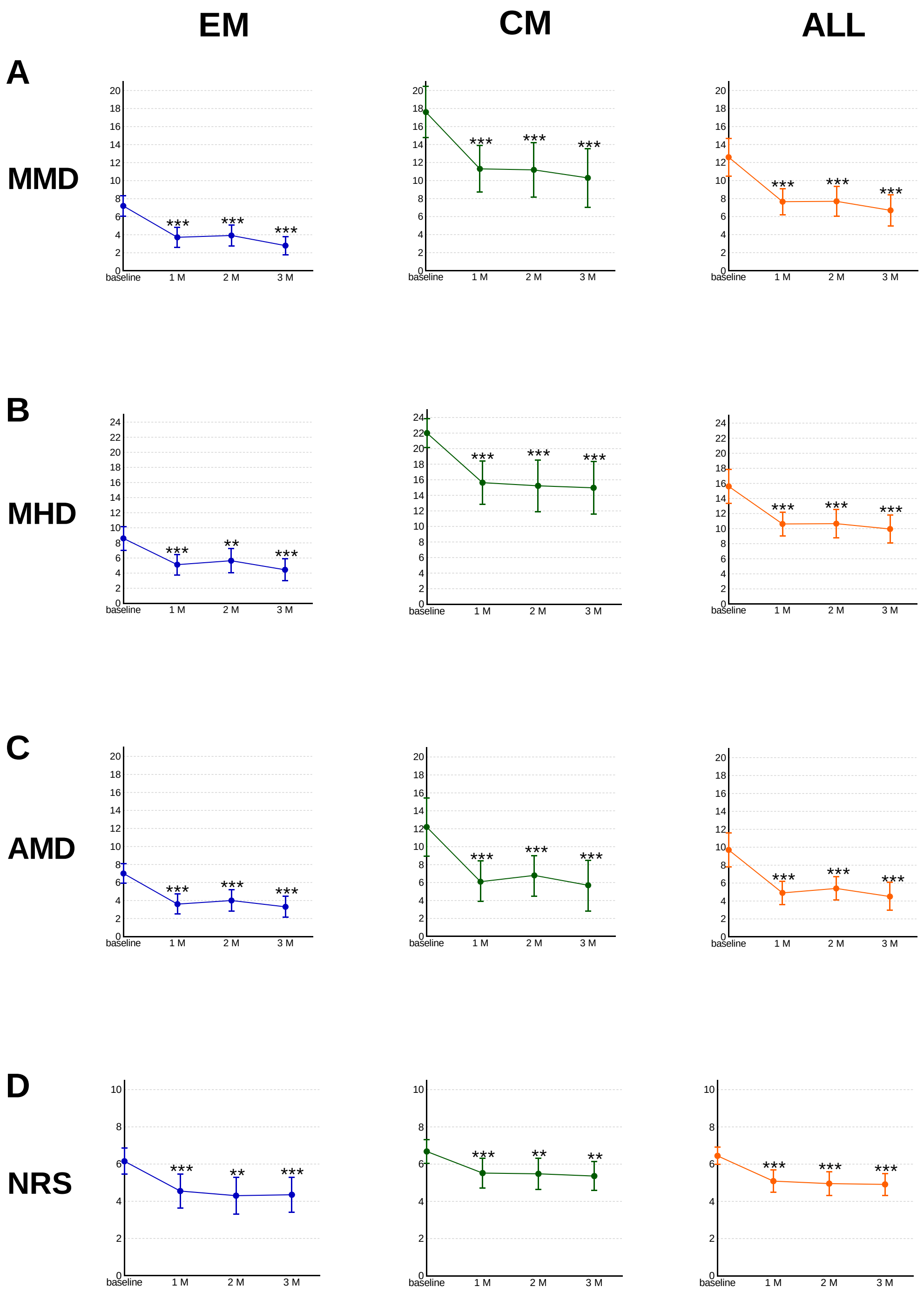

Supplementary Figure 1. Changes in (A) MMD, (B) MHD, (C)AMD, and (D) NRS

～* represents a statistically significant change. **adjusted p<0.01; ***adjusted p<0.001

Line graphs are expressed as mean and bars represent 95% CI.

MMD, monthly migraine day; MHD, monthly headache day; AMD, monthly acute medication intake days; NRS, numerical rating scale; EM, episodic migraine; CM, chronic migraine; ALL, all patients; 1 M, 1 month; 2 M, 2 months; 3 M, 3 months
