# Supplementary material for: Real-world evidence of galcanezumab for migraine treatment in Japan: a retrospective analysis
Source: BMC Neurol. 2022 Dec 31;22:512. doi: 10.1186/s12883-022-03041-1 (PMC9805082; doi:10.1186/s12883-022-03041-1)
Supplement: Supplementary file 2 — Additional file 2: Supplementary Figure 2. Fifty percent responder rate in patients with or without medication-overuse headache. MOH: Medication-overuse headache. Responder rate was based on monthly migraine days. [file 12883_2022_3041_MOESM2_ESM.pdf]

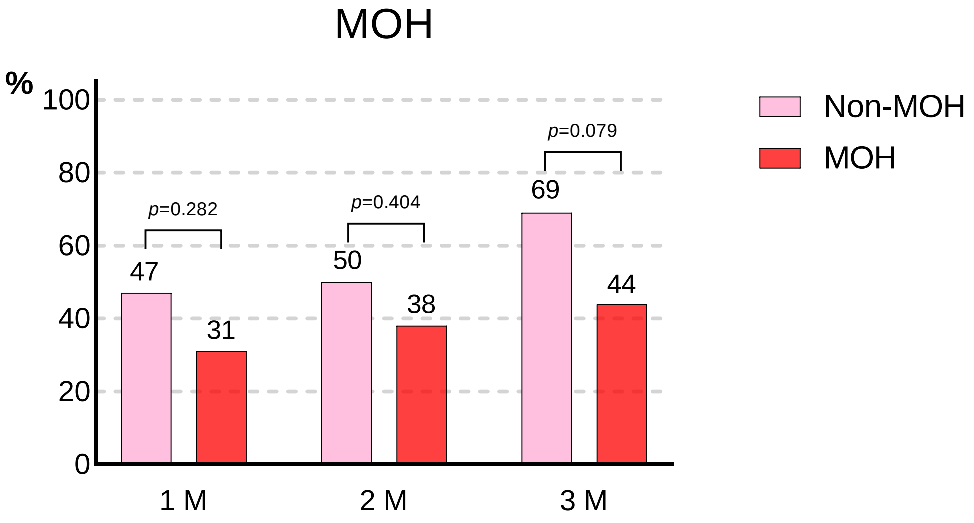

Supplementary Figure 2. Fifty percent responder rate in patients with or without medication-overuse headache

MOH: Medication-overuse headache

Responder rate was based on monthly migraine days.
