# Supplementary material for: Real-world evidence of galcanezumab for migraine treatment in Japan: a retrospective analysis
Source: BMC Neurol. 2022 Dec 31;22:512. doi: 10.1186/s12883-022-03041-1 (PMC9805082; doi:10.1186/s12883-022-03041-1)
Supplement: Supplementary file 3 — Additional file 3: Supplementary Figure 3. Fifty percent responder rate in patients in GAD-7 < 5 and GAD-7 ≥ 5 (anxiety). GAD-7: Generalized Anxiety Disorder-7. Responder rate was based on monthly migraine days. [file 12883_2022_3041_MOESM3_ESM.pdf]

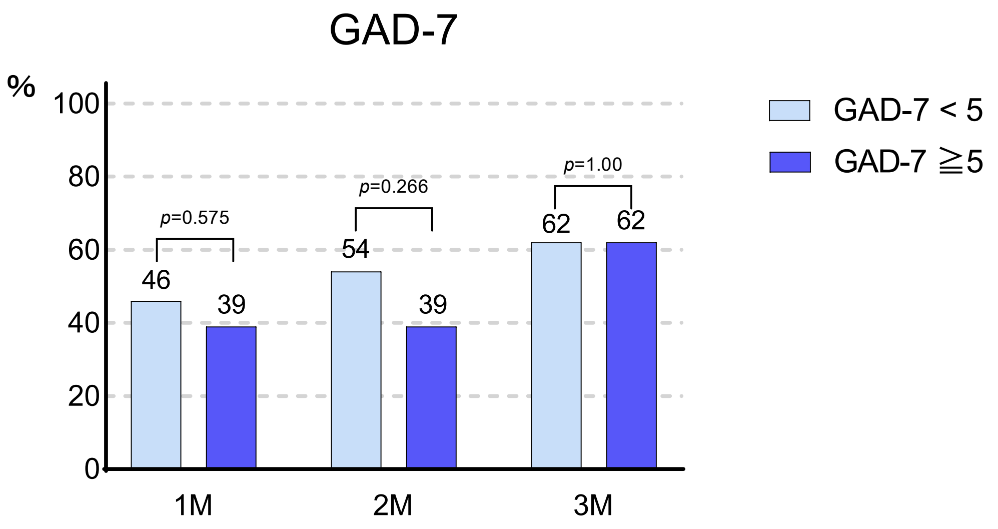

Supplementary Figure 3. Fifty percent responder rate in patients in GAD-7 < 5 and GAD-7 ≥ 5 (anxiety).

GAD-7: Generalized Anxiety Disorder-7

Responder rate was based on monthly migraine days.
