# Supplementary material for: Real-world evidence of galcanezumab for migraine treatment in Japan: a retrospective analysis
Source: BMC Neurol. 2022 Dec 31;22:512. doi: 10.1186/s12883-022-03041-1 (PMC9805082; doi:10.1186/s12883-022-03041-1)
Supplement: Supplementary file 4 — Additional file 4: Supplementary Figure 4. Degree of associated symptoms at baseline and after 1-3 M of galcanezumab. (A) Photophobia, (B) Phonophobia, and (C) Nausea/vomiting. 1 M: 1 month, 3 M: 3 months. [file 12883_2022_3041_MOESM4_ESM.pdf]

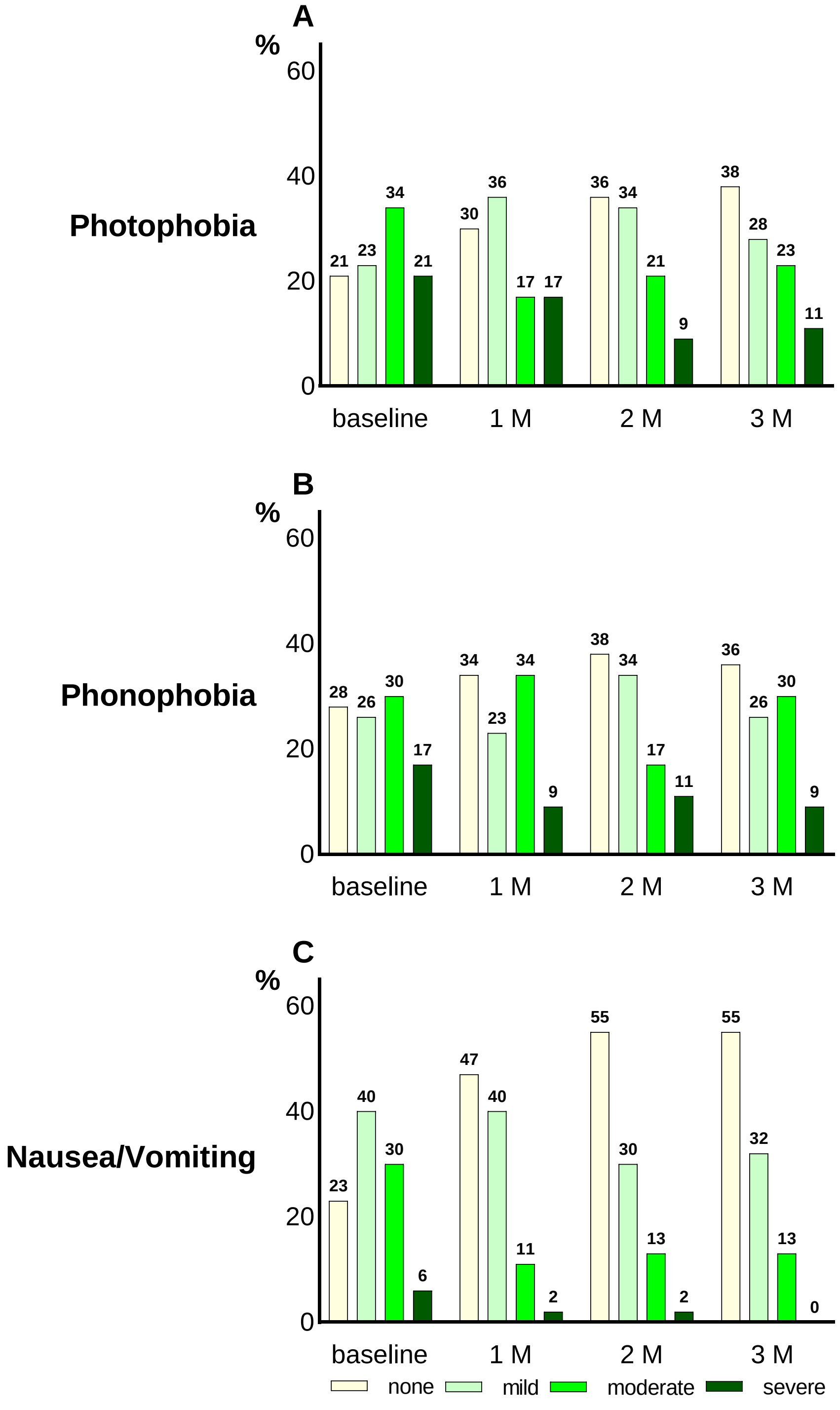

Supplementary Figure 4. Degree of associated symptoms at baseline and after 1-3 M of galcanezumab

(A) Photophobia, (B) Phonophobia, and (C) Nausea/vomiting

1 M: 1 month, 3 M: 3 months
