# Supplementary material for: Real-world evidence of galcanezumab for migraine treatment in Japan: a retrospective analysis
Source: BMC Neurol. 2022 Dec 31;22:512. doi: 10.1186/s12883-022-03041-1 (PMC9805082; doi:10.1186/s12883-022-03041-1)
Supplement: Supplementary file 5 — Additional file 5: Supplementary Figure 5. Improvement and disappearance in associated symptoms in patients with or without 50% responder rate in MMD. (A) Photophobia, (B) Phonophobia, and (C) Nausea/vomiting non-Res: non-responder (<50% responder rate), Res: responder (≥50% responder rate), MMD: monthlymigraine days. Responder rate was based on monthly migraine days. [file 12883_2022_3041_MOESM5_ESM.pdf]

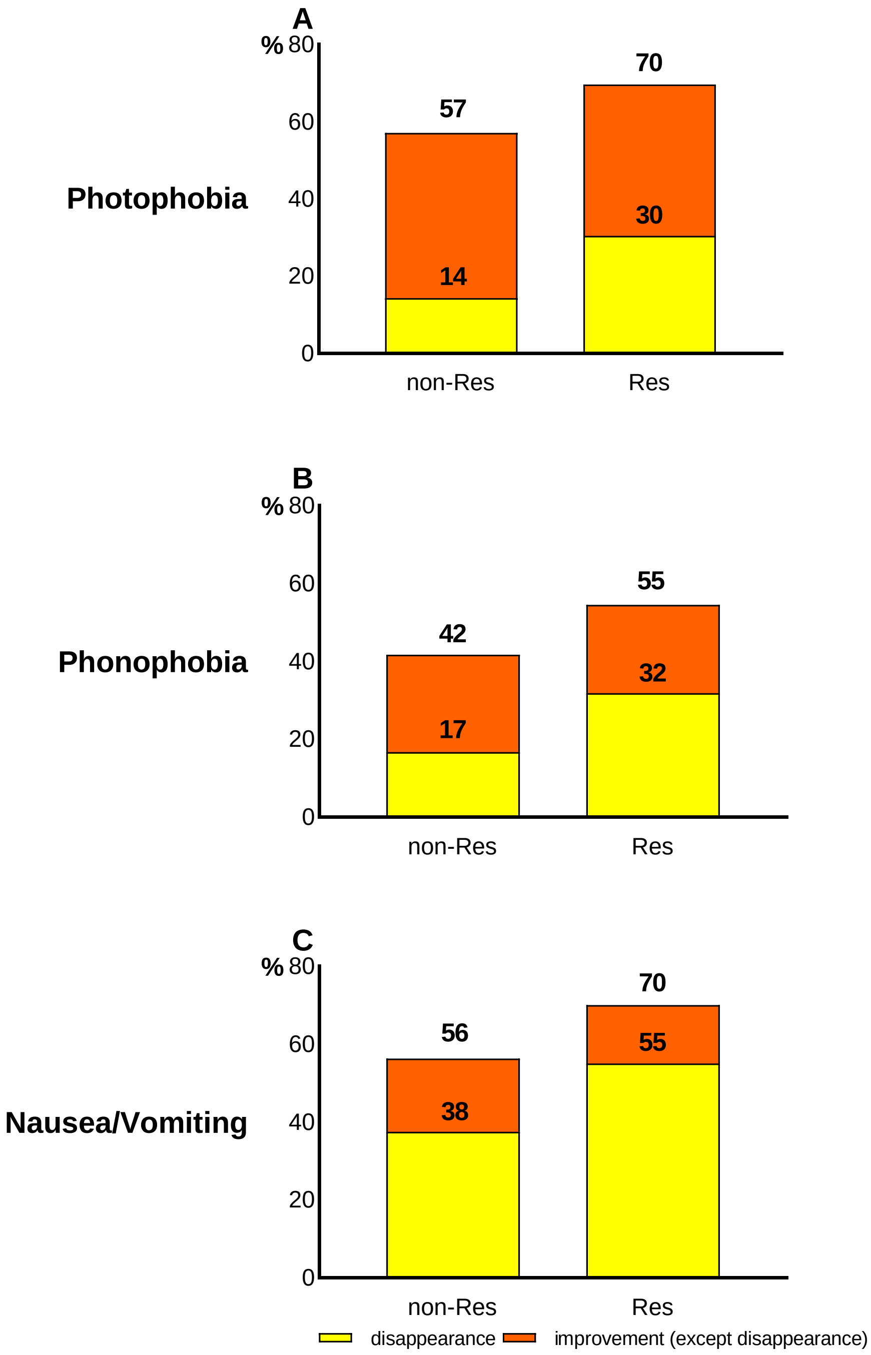


Supplementary Figure 5. Improvement and disappearance in associated symptoms in patients with or without 50% responder rate in MMD.

(A) Photophobia, (B) Phonophobia, and (C) Nausea/vomiting

non-Res: non-responder (<50% responder rate), Res: responder (≥50% responder rate), MMD: monthly migraine days.

Responder rate was based on monthly migraine days.
